# Evaluation of the pooled sample method in Infinium MethylationEPIC BeadChip array by comparison with individual samples

**DOI:** 10.1186/s13148-023-01544-3

**Published:** 2023-08-28

**Authors:** Shota Nishitani, Takashi X. Fujisawa, Akiko Yao, Shinichiro Takiguchi, Akemi Tomoda

**Affiliations:** 1 Research Center for Child Mental Development, University of Fukui, Fukui, Japan; 2 Division of Developmental Higher Brain Functions, United Graduate School of Child Development, Osaka University, Kanazawa University, Hamamatsu University School of Medicine, Chiba University, University of Fukui, Osaka, Japan; 3 Life Science Innovation Center, University of Fukui, Fukui, Japan; 4 Department of Child and Adolescent Psychological Medicine, University of Fukui Hospital, Fukui, Japan

**Keywords:** Blood, Child, DNA methylation, Epigenome, Illumina EPIC array, Methodology, Pooled sample, Pooling

## Abstract

**Background:**

The pooled sample method is used in epigenomic research and expression analysis and is a cost-effective screening approach for small amounts of DNA. Evaluation of the pooled sample method in epigenomic studies is performed using the Illumina Infinium Methylation 450K BeadChip array; however, subsequent reports on the updated 850K array are lacking. A previous study demonstrated that the methylation levels obtained from individual samples were accurately replicated using pooled samples but did not address epigenome-wide association study (EWAS) statistics. The DNA quantification method, which is important for the homogeneous mixing of DNA in the pooled sample method, has since become fluorescence-based, and additional factors need to be considered including the resolution of batch effects of microarray chips and the heterogeneity of the cellular proportions from which the DNA samples are derived. In this study, four pooled samples were created from 44 individual samples, and EWAS statistics for differentially methylated positions (DMPs) and regions (DMRs) were conducted for individual samples and compared with the statistics obtained from the pooled samples.

**Results:**

The methylation levels could be reproduced fairly well in the pooled samples. This was the case for the entire dataset and when limited to the top 100 CpG sites, consistent with a previous study using the 450K BeadChip array. However, the statistical results of the EWAS for the DMP by individual samples were not replicated in pooled samples. Qualitative analyses highlighting methylation within an arbitrary candidate gene were replicable. Focusing on chr 20, the statistical results of EWAS for DMR from individual samples showed replicability in the pooled samples as long as they were limited to regions with a sufficient effect size.

**Conclusions:**

The pooled sample method replicated the methylation values well and can be used for EWAS in DMR. This method is sample amount-effective and cost-effective and can be utilized for screening by carefully understanding the effective features and disadvantages of the pooled sample method and combining it with candidate gene analyses.

**Supplementary Information:**

The online version contains supplementary material available at 10.1186/s13148-023-01544-3.

## Background

Research on epigenomes and their interaction with genetics that reflect the “nurture and environment” aspect has shifted focus from solely studying genetic sequences in various fields [1]. Epigenomic research, initially dominated by cancer research [2], has recently expanded to include studies on the relationship between various phenotypes, such as aging [3–7], nutrition and diet [8], environmental pollutants [9, 10], immunity [11, 12], neurological disorders [13], psychiatric disorders [14–17], and psychological traits [18, 19], and is expected to increase further [20].

Converse to data-driven genome-wide association analysis, hypothesis-driven candidate gene analysis is the primary approach for conducting epigenomic studies. However, studies that focus on the results of candidate gene analysis and candidate gene-by-environment interactions alone for understanding complex traits without replication experiments are becoming outdated [21]. As genome-wide data, including next-generation sequencing and microarrays, have become widely available, epigenomic research is now dominated by genome-wide association analyses [22].

However, microarrays, the gateway for genome-wide association analysis, can cost several hundred dollars per sample. Given the need for a sample size as large as possible to narrow down certain results from genome-wide association analysis, barriers to the entry of beginners into epigenomic research are very high. This cost is likely a barrier especially for researchers in related fields outside the biomedical sciences and can potentially cause a lack of expansion in the scope of epigenomic research.

These circumstances lead to the use of the pooled sample method [23–25]. The pooled sample method combines several samples from the same experimental group equally into a few samples, with samples from a control group prepared similarly, and compares them, thereby reducing the cost of genome-wide analysis. This method has long been used in epigenomic research, as well as in expression analysis, and is cost-effective as a screening approach [26, 27]. The pooled method has been validated in epigenomic studies using the Illumina Infinium Methylation 450 K BeadChip array [23]; however, there have been no subsequent reports. A previous study only demonstrated that the β values, which reflect methylation levels, obtained from individual samples were accurately replicated using pooled samples and did not address the epigenome-wide association study (EWAS) statistics estimated using pooled samples. The goal of genome-wide association studies is to narrow down the CpG sites and genes associated with a variable of interest from the genome-wide data; however, it is not clear from the results of the above study whether this is possible with pooled samples. Although the Illumina methylation array has been updated from 450 to 850K (EPIC), no study has validated the pooled sample method based on the Illumina Infinium MethylationEPIC BeadChip array. The pooled sample method combines samples equally and requires precise adjustment of concentrations. Spectrofluorimetry is the mainstream method for quantifying DNA concentrations because of its precision [20]. The problems of batch effects depending on the chip and row layout of the microarray have also been raised [28–31]. Recent studies have attempted to account for cellular heterogeneity in the DNA extracted for methylation analysis using a data science method for the microarray data [32], in contrast to target analysis, which cannot address this issue. Therefore, an update is necessary to validate the pooled sample method.

In the present study, we evaluated the validity of the pooled sample method following the validation protocol by a previous study conducted at 450K with EPIC and adding the following new elements: comparison of EWAS statistics of differential methylation positions (DMPs) between individual and pooled samples, comparison of qualitative characteristics when focusing on an arbitrary candidate gene, and comparison of EWAS statistics of differentially methylated regions (DMRs) between individual and pooled samples.

## Results

### Correlation analysis for β- and M-values in the full dataset (820,677)

**Fig. 1:**
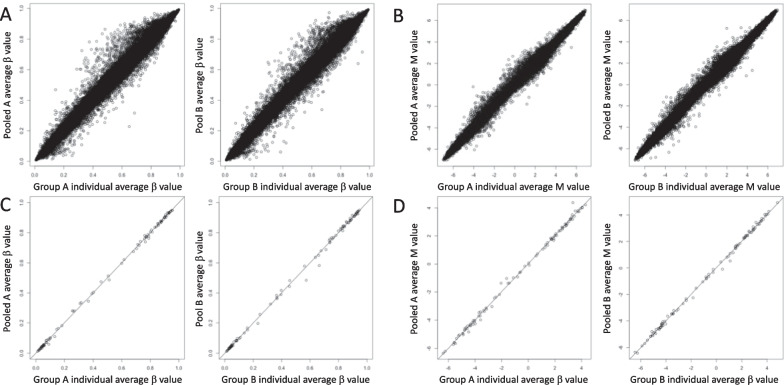
Correlation plots for (**A** and **B**) the full dataset and (**C** and **D**) 100 DMPs between averaged *β*-values (**A** and **C**) and M-values (**B** and **D**) of individual and pooled samples. Left: correlations for Group A. Right: correlations for Group B. The X-axis represents the average methylation value for individual samples, and the Y-axis represents the average methylation value for pooled samples

The average values of the individual samples included in each pool and the average values of each pooled sample showed a robust correlation (group A: [*β*-value] *rho* = 0.998, *P* = 2.20E−16, [*M*-value] *rho* = 0.998, *P* = 2.20E−16; group B: [*β*-value] *rho* = 0.997, *P* = 2.20E−16, [*M*-value] *rho* = 0.998, *P* = 2.20E−16) (Fig. 1A and B).

### One sample *t* tests for each pooled sample against the range of methylation levels in the individual sample group

Almost half the probes of the value from the pooled sample were out of the range when applying the nominal significant level (Additional file 1: Table S1). When multiple comparisons were corrected in the Benjamini–Hochberg (BH) false discovery rate (*FDR*), about 40% were significant, indicating that many probes were outside the range of the distribution of individual samples. However, for the Bonferroni correction, it was less than 1%, indicating that most of the probes were within the range; thus, the correction method used makes a substantial difference in interpretation. In addition to these corrections, a threshold of 9.0 × 10^−8^ has been proposed as a significant threshold for genome-wide analysis using the Illumina EPIC array [33]. Using such stringent thresholds (9.0E-08 or Bonferroni), which are not prone to type I errors, most sites in the pooled samples would have been within the range of distribution for individual samples. This result was similar for both *β*- and *M*-values (Additional file 1: Table S1).

### EWAS for the experimental group using individual samples

The EWAS for the experimental group was conducted using regression analyses without adding covariates and performed using *CpGassoc* [34]. The results showed that 254 [*β*-value] and 256 [*M*-value] DMPs were significantly associated with the experimental group at BH *FDR* < 0.05 (*β*-value: 1.97E−09 < *Ps* < 1.54E−05 and *M*-value: 1.19E−09 < *Ps* < 1.56E−05). However, because the present analysis did not adjust for covariates, the *Q*–*Q* plot revealed a slightly inflated lambda of 1.4 (*β*-value) and 1.43 (*M*-value). This may lead to Type I errors. Therefore, we decided to use the top 100 CpGs (*β*-value: 1.97E−09 < *Ps* < 4.09E−06 and *M*-value: 1.19E−09 < *Ps* < 4.07E−06) for subsequent analyses rather than those supported by the statistical results.

### Correlation analysis for β-values using the top 100 DMPs

The average values for the top 100 DMPs from the EWAS analysis using individual samples and the average values for the top 100 DMPs of each pooled sample showed a robust correlation (group A: [*β*-value] *rho* = 0.998, *P* = 2.20E−16, [*M*-value] *rho* = 0.998, *P* = 2.20E−16; group B: [*β*-value] *rho* = 0.998, *P* = 2.20E−16, [*M*-value] *rho* = 0.998, *P* = 2.20E−16) (Fig. 1C and D).

### Correlation analysis for EWAS statistics from the entire DMP analysis

**Fig. 2:**
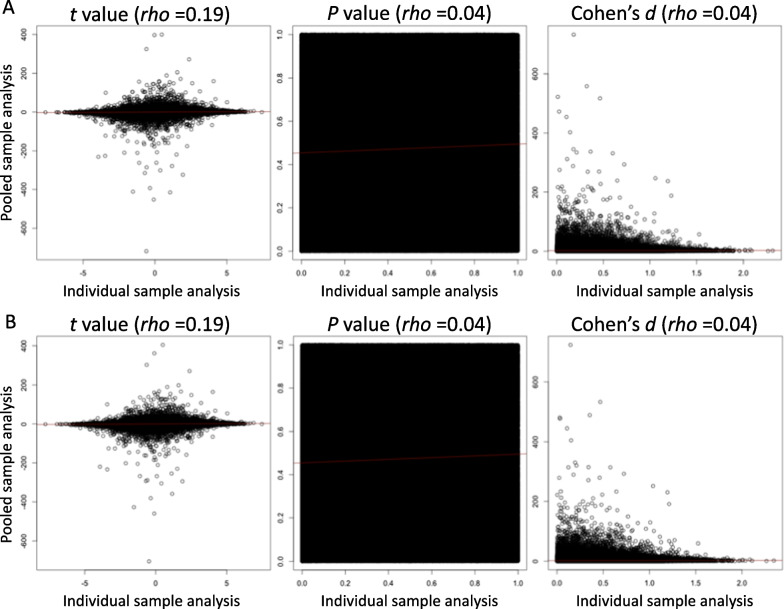
Correlation plots for EWAS statistics of differential methylation position (DMP) analysis between individual and pooled samples. **A** *β*- and **B** *M*-value

The EWAS statistics of DMP analysis conducted for the pooled sample could not replicate those obtained using individual samples ([*β*-value]* t* value: *rho* = 0.19, *P *value = 2.2E−16, *P *value: *rho* = 0.04, *P* value = 2.2E−16, and Cohen’s *d*: *rho* = 0.04, *P *value = 2.2E−16, [*M*-value]* t* value: *rho* = 0.19, *P* value = 2.2E−16, *P* value: *rho* = 0.04, *P* value = 2.2E−16, and Cohen’s *d*: *rho* = 0.04, *P* value = 2.2E−16) (Fig. 2).

### Qualitative comparisons in an arbitrary candidate gene

**Fig. 3:**
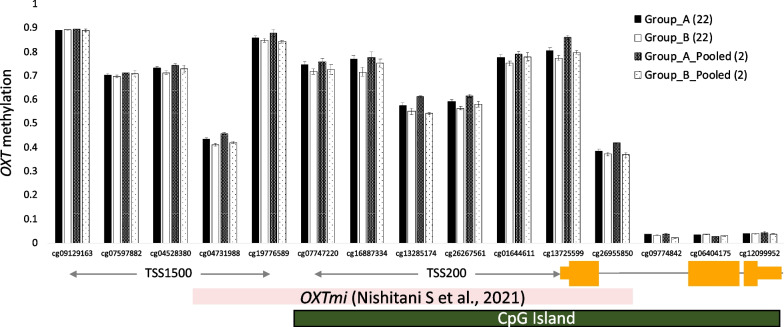
Comparison between averaged β-values of individual and pooled samples for 15 CpG sites in the *OXT* gene. The error bar represents standard error. Values in parentheses indicate sample numbers. The figure at the bottom shows the genetic features. TSS: transcription start site

The bar plot in Fig. 3 shows the same trend for individual and pooled samples, with = 13/15 (86.7%) coincidence in the high or low direction. Other results of the pooled samples are available on this website (https://lookerstudio.google.com/u/0/reporting/7f4664a5-0c11-4bbd-ac02-2a9b19eee38b/page/mR7OD).

### Correlation analysis for EWAS statistics from DMR analysis for chr 20

**Fig. 4:**
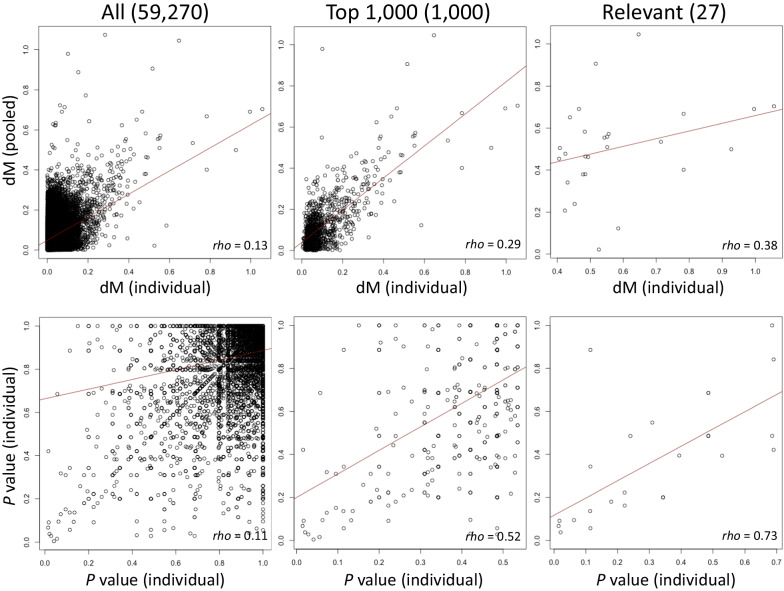
Correlation plots for EWAS statistics of differential methylation region (DMR) analysis between individual and pooled samples

**Fig. 5:**
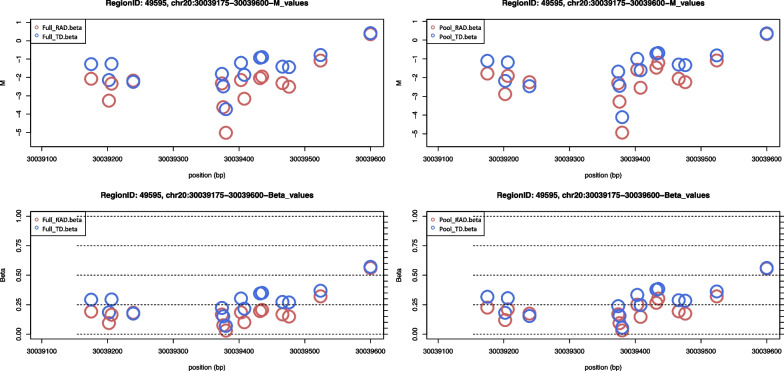
Top 1 relevant DMR plot comparing the result from individual (Left) and pooled samples (Right)

The EWAS statistics from the DMR analysis on chr 20 conducted on the pooled sample partially replicated the results obtained from individual samples (Fig. 4). When all DMRs (59,270) were used, the reproducibility of the results was very low (median difference between *M*-values in a region: dM: *rho* = 0.13, *P* value = 2.20E−16; *P* value: *rho* = 0.11, *P* value = 2.20E−16). When limited to the top 1000 DMRs, the results were more or less the same (dM: *rho* = 0.52, *P* value = 2.20E−16 *P* value; *rho* = 0.29, *P* value = 2.20E−16). Furthermore, the results were substantially replicable when limited to DMRs that met the relevant conditions (d*M* > 0.4) recommended in *DMRforPair* (d*M*: *rho* = 0.38, *P* value = 0.053, *P* value: *rho* = 0.73, *P* value = 1.83E−05) [35]. Twenty-seven and 75 relevant DMRs were identified from the *DMRforPair* analysis using individual and pooled samples, respectively (Additional file 2). Of the 27 relevant DMRs, 19 were included in the 75 DMRs found in the pooled samples (Additional file 2). The top-most relevant DMR found from individual sample analysis was compared to the results from the pooled sample as an example (Fig. 5).

## Discussion

In this study, we evaluated the usefulness of the pooled sample method in EWAS. This is the first validation using the Illumina Infinium MethylationEPIC BeadChip array (850K), nearly doubling the amount of data used in a previous study. The results showed that, as in a previous study [23], even at 850K, the *β*-values of individual and pooled samples were robustly correlated, both for the complete data set and the top 100 DMPs from EWAS. This indicates that the pooled samples replicated the mean *β*-values quite well. Although the pooled samples were highly replicable in terms of *β*-values, the EWAS statistics of the DMP analysis conducted on the pooled samples did not replicate the results obtained for individual samples suggesting that data obtained from pooled samples should not be used to make statistical interpretations for DMP analysis. This should be avoided because of the risk of dissociation from the results of analysis using individual samples, which could lead to distorted interpretations. In the present study, the number of pooled samples were set at two, and although increasing the number of pooled samples may improve reproducibility, this would not be a cost-effective way to achieve the goal. Thus, it is necessary to consider how to reasonably use data obtained using the pooled method with limited samples in cases other than DMP analysis.

We focused on the *OXT* gene as an arbitrary candidate and compared the *β*-values of the CpG sites between individual and pooled samples. The results, although qualitative, showed that 13 of the 15 sites (86.7%) were consistent in the direction of high or low. Our previous study reported that nine CpGs were densely located in the promoter region of this gene and can be considered as a single factor (*OXT*mi) based on the results of factor analysis [14]. Although the implications of the results from the experimental groups are not directly relevant to the present study, the DNA methylation levels of the nine CpG sites included in *OXT*mi were consistently higher in group A, which corresponds to the RAD group, than in group B, which corresponds to the TD group, in line with the previous study [14]. The populations in the present study did not exactly match those in the previous study; therefore, this was not a comparison between the same populations. Nevertheless, the trend that *OXT*mi was higher in maltreated children diagnosed as reactive attachment disorder (RAD) than in typically developing (TD) children was also observed in the pooled samples, suggesting that the pooled samples replicated the results from individual samples and the previous study. Although we highlighted the *OXT* gene, it is expected that other genes can also be used for qualitative assessment. We have created a web database where the entire results from the pooled samples can be viewed to enable such qualitative analysis (https://lookerstudio.google.com/u/0/reporting/7f4664a5-0c11-4bbd-ac02-2a9b19eee38b/page/mR7OD).

Since we found that the EWAS statistics of the DMP analysis conducted on the pooled samples did not replicate the results obtained from individual samples, the only alternative option was to conduct a DMR analysis using the R package, *DMRforPairs*, which can perform a one vs. one comparison. This is a nonparametric analysis that tests the sequential high or low of four or more consecutive CpG sites using the Mann–Whitney U test so that even one vs. one statistical analysis can be performed. In fact, *DMRforPairs* analysis of chr 20 showed that the results of the individual and pooled samples were reasonably correlated if we focused on the relevant DMRs. Of the 27 relevant DMRs identified in the individual sample analysis, 19 (70.4%) DMRs were replicated with statistical support in the pooled samples. These results suggest that, with filtering that excludes DMRs with low dM (i.e., low effect sizes) between the two groups [36], the EWAS statistics of DMR from pooled samples may yield results similar to those of individual samples. However, significant DMRs obtained in the present analysis were only three of 27 relevant DMRs at the uncorrected 5% level (*P* < 0.05), and it is worth investigating whether this one vs. one method can yield robust *P* values that can survive multiple corrections. To conduct this analysis, the *β*-values of individual samples were averaged to make them correspond to a single value. This manipulation may cause dissociation or differences in detection rates compared to results of conventional DMR analysis conducted on the original sample size. Despite these limitations, it may be useful to conduct *DMRforPairs* analysis on pooled samples and limit the focus to regions with d*M* > 0.4, because it reproduced the results of individual samples. However, the difference of d*M* > 0.4 in the *M*-value scale can be translated to the difference of up to ~ 0.07 in the *β*-value scale, which is indeed small. Although d*M* > 0.4 was used in the present study to show multiple DMR listings as an example, the threshold of |d*M*| should be higher.

The pooled sampling method has several advantages. The first is that β-values are well replicated, whether for the full set, the top 100, or a candidate gene. Thus, this method would be suitable for qualitative examination of methylation levels. Second, microarrays can be used to estimate cell proportions even in the case of pooled samples, in contrast to target analysis methods such as pyrosequencing and MALDI-TOF/MS EpiTYPER^®^. Third, the pooled method is useful when the amount of DNA from an individual sample alone is insufficient for DNA methylation profiling, such as when using rare or old samples. Fourth, undoubtedly, it can be achieved on a low budget. In our case, the cost would be $1200 for the four pooled samples and $13,200 for the 44 individual samples if the microarray for each sample costs $300. This is approximately a tenfold difference in price and may encourage the introduction of genome-wide analysis to entry-level scientists or novice researchers in related fields. Fifth, some journals no longer accept studies that are based only on hypothesis-driven candidate genes or gene-by-environment interactions for studies of complex traits, without replication experiments [21]. Several hypothesis-driven studies have been published that examine the correspondence between SNPs and methylation within a candidate gene and some phenotypes. However, now that data-driven genome-wide analyses have become mainstream, some journals have begun to discourage the method of examining only candidate genes, because it does not eliminate researcher bias and is unlikely to lead to new discoveries. In such an analysis, the requirement for the replication of experiments within the same study and the requirement for genome-wide significance levels has raised the standard for the acceptance of manuscripts. The pooled sample method alone may not be sufficient to narrow down genome-wide associations owing to statistical disadvantages; however, in combination with candidate analysis, it may provide more than the required level of evidence by the journals.

## Conclusions

The present study is the first to use the pooled sample method utilizing the 850K (EPIC) array for blood samples from children. It will be challenging to implement genome-wide analysis as it requires expertise in areas such as bioinformatics and a large budget. Despite its statistical disadvantages, the pooled sample method may be useful for expanding the scope of epigenomic research.

## Methods

### Subjects and construction of the pooled sample

**Fig. 6:**
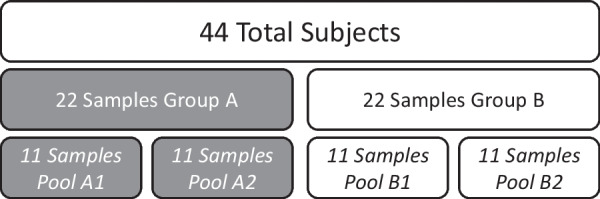
Pooled sample composition. Forty-four subjects were included in this study, 22 of them from Group A and 22 from Group B. Two pools were created from group A, with 11 samples each (Pool_A1 and Pool_A2), and two more pools of 11 samples were created from group B (Pool_B1 and Pool_B2)

In total, 44 baseline blood samples from Japanese male children (13.1 ± 1.7 years) from a part of our previous clinical trial study (UMIN-CTR; UMIN000013215) were used [37]. Of these 44 participants, 22 were children diagnosed with reactive attachment disorder (RAD) (group A). All participants with RAD had a history of child maltreatment, including physical abuse, neglect, emotional abuse, and sexual abuse. Age-matched 22 typically developing children (group B) recruited from the general population were used as controls. Two pooled samples were constructed using the samples from groups A (Pool_A1 and Pool_A2) and B (Pool_B1 and Pool_B2) (Table 1 and Fig. 6). Pool_A1 (12.9 ± 1.8 years) and Pool_A2 (13.1 ± 1.9 years) were age-matched with Pool_B1 (13.0 ± 1.4 years) and Pool_B2 (13.5 ± 2.0 years), respectively (Additional file 1: Fig. S1).

**Table 1:** Population and pooled sample characteristics

	Pool_A1	Pool_A2	Pool_B1	Pool_B2
*n*	11	11	11	11
Age (years)	12.9 ± 1.8	13.2 ± 2.2	13.0 ± 1.4	13.4 ± 1.6
Male/female	11/0	11/0	11/0	11/0
DNA concentration (ng/μL)				
Spectrofluorimetry	14.5 ± 1.4	15.0 ± 1.1	15.3 ± 1.0	15.2 ± 1.4
Spectrophotometry	19.8 ± 2.2	21.6 ± 2.7	20.9 ± 2.4	23.0 ± 3.3
260/280 ratio	1.86 ± 0.06	1.86 ± 0.04	1.84 ± 0.05	1.83 ± 0.08

### DNA extraction and pooled sample preparation

Whole blood samples were collected in EDTA tubes and immediately preserved in RNAlater® (whole blood: RNAlater^®^ = 5:13). They were stored overnight at 4 °C and then at − 20 °C for long-term storage. Although this preprocessing method is that originally used for RNA extraction, subsequently we followed the instructions specified in the RiboPure™ RNA Purification Kit (Thermo Fisher Scientific, Inc., MA, US) for DNA extraction using RNAlater®-preserved blood samples [38]. Briefly, RNAlater^®^-preserved blood samples (720 μL) were centrifuged for 1 min at 16,000×*g* and the supernatant were removed. Next, 200 μL of PBS was added and mixed, and the sample was centrifuged. The supernatant removal was repeated. Finally, 200 μL of PBS was added and mixed by pipetting before the samples were used for DNA extraction. The QIAamp DNA Blood Mini Kit (QIAGEN, Hilden, Germany) was used to extract DNA. The DNA yield was determined using a Qubit™ dsDNA High Sensitivity Assay Kit (Thermo Fisher Scientific, Inc., MA, US) [20]. Following previous protocols for handling pooled samples [23–25], dilutions were made using TE buffer to obtain a concentration of 15 ng/μl for each sample, accurately diluted to within ± 5 ng/μl preciseness. The final concentration of each sample is summarized in Additional file 3. As previous studies have used UV photospectrometry, the final diluted samples were measured using a NanoDrop 2000 (Thermo Fisher Scientific, Inc., MA, US), and the results were summarized. Individual DNA samples were then added to their respective pool (10 μl of 15 ng/μl sample). Once each pool was generated, the DNA concentrations were re-quantified with Qubit™ and NanoDrop to ensure that the final concentration of the pool was as expected (15 ng/μl based on Qubit™; Additional file 4). Only when the final pool concentration was 15 ± 5 ng/μl and the total volume was 110 μl as expected, it was used for the next step.

### Preprocessing

For each sample, 500 ng of DNA (44 individual samples and four pooled samples) was bisulfite-converted using the EZ DNA Methylation™ Kit (Zymo Research, D5002). An Infinium HumanMethylationEPIC BeadChip Kit (Illumina, WG-317-1002) array was used to assess genome-wide DNA methylation. Most importantly, samples were grouped by individuals and balanced onto chips to avoid confounding effects generated by batch processing [28–31] (Additional file 1: Fig. S2). The arrays were scanned using the Illumina iScan platform. The methylation level (*β*-value) was calculated using the R package *minfi* [39], followed by a Psychiatric Genomics Consortium-Epigenome-Wide Association Studies quality control (QC) pipeline [16]. We used the R package, *CpGassoc* [34], to filter out samples with probe detection call rates < 90% and an average intensity value of either < 50% of the experiment-wide sample mean or < 2000 arbitrary units (AU). We set low-quality probes (detection *P* values > 0.01) as missing and filtered out probes that were missing for > 10% of samples. Probes containing single nucleotide polymorphisms (SNPs; based on 1000 Genomes) within ten base pairs of the target CpG were maintained. Cross-hybridizing probes were removed [40]. A total of 820,677 probes that passed the QC were included in our analyses. We performed single sample Noob (ssNoob) normalization using *minfi* [39]. To remove the chip and positional batch effects, we applied ComBat (R package *sva*) [41] to protect group status [30]. This decision was made because the singular value decomposition calculated by the R package *ChAMP* [42] revealed the confounding effects of unwanted non-biological variables, despite setting a balanced layout for the experimental group (Additional file 1: Fig. S3). For each sample, including the pooled samples, cellular heterogeneity (i.e., the proportion of CD8 + T cells, CD4 + T cells, natural killer (NK) cells, B cells, monocytes, and neutrophils) was predicted using the robust partial correlation method implemented in the *EpiDISH* method [32] using the reference data reported previously [43] (Additional file 1: Table S2).

### Statistical analysis

The accuracy of the DNA methylation-level estimations from the pooled DNA was assessed using Spearman’s correlation between the average values of the individual samples included in each pool and the average values of each pooled sample. These evaluations were conducted for both *β*- and *M*-values; the correlation analysis was conducted for both the full dataset and the top 100 DMPs found in the EWAS using individual samples (Group A vs. Group B). To further assess the replicability of *β*- and *M*-values in each pooled sample (A1, A2, B1, and B2), one sample *t* tests were conducted against the range of methylation level in the individual sample group (A1:11, A2:11, B1:11, and B2:11) for 820,677 sites. The number of significant sites was regarded as out of range for each of the four criteria when considering the multiple-comparison corrections (BH *FDR*, 9.0E-08, and Bonferroni corrections) in addition to the uncorrected. To evaluate the replicability of the statistical analysis for DMPs, a single regression model was applied to the 820,677 sites using *CpGassoc* [34] with the group as the independent variable and methylation as the dependent variable for individual and pooled samples. The *t* values, *P* values (uncorrected), and Cohen’s *d*-values obtained for each case were evaluated using Spearman’s correlation analysis. To demonstrate the accuracy of DNA methylation-level estimations from pooled DNA for a given candidate gene, we extracted data from 15 CpG sites on the *OXT* gene as a representative and plotted the averaged values for qualitative comparison. To evaluate the replicability of the statistical analysis for DMR, we selected chr 20 as the arbitral target. The DMR analysis was conducted using the R package, *DMRforPair* [35]. Because this was a one vs. one analysis, we used the mean of each of the individual and pooled sample groups. We set “min_n” as 4 since the Mann–Whitney U test requires a minimum of seven samples to ever reach a *P* value < 0.05 (2 groups × 4 CpGs = 8). The other parameters were set as follows: 200 for “min_distance” for the rapid drop of co-methylation of adjacent probes when they were further apart [44, 45], and 0.4 for “min_dM” due to minimal recommendation [36] when we limited relevant DMRs at the cost of allowing more false-positives. The dM and *P* values obtained for each case were evaluated using Spearman’s correlation analysis. Default values were used for other parameters. This correlation analysis was conducted for all regions (59,270), the top 1000 regions found in the DMR analysis using individual samples (Group A vs. Group B), and the relevant DMRs (d*M* > 0.4) found in the individual sample analysis. All statistical analyses were performed using R software (version 4.2.1) [46].

### Supplementary Information

**Additional file 1**. Supplementary Table S1, Supplementary Figure S1, Supplementary Figure S2, Supplementary Figure S3, Supplementary Table S2

**Additional file 2**. Supplementary Dataset 1

**Additional file 3**. Supplementary Dataset 2

**Additional file 4**. Supplementary Dataset 3

## Data Availability

The DNA methylation microarray data for pooled samples that support the study findings have been deposited in the Gene Expression Omnibus database (GEO) under the primary accession code GSE231532. Results of the pooled samples are available on this website (https://lookerstudio.google.com/u/0/reporting/7f4664a5-0c11-4bbd-ac02-2a9b19eee38b/page/mR7OD). Further inquiries can be directed to the corresponding author.

## Abbreviations

EWAS: Epigenome-wide association study
DMPs: Differential methylated positions
DMRs: Differentially methylated regions
d*M*: Median difference between *M*-values in a region
MALDI-TOF/MS: Matrix-assisted laser desorption/ionization-time of flight/mass spectrometry
ICD-10: International Classification of Diseases 10th Revision
RAD: Reactive attachment disorder
