# Supplementary material for: Evaluation of the pooled sample method in Infinium MethylationEPIC BeadChip array by comparison with individual samples
Source: Clin Epigenetics. 2023 Aug 28;15:138. doi: 10.1186/s13148-023-01544-3 (PMC10463626; doi:10.1186/s13148-023-01544-3)
Supplement: Supplementary file 1 — Additional file 1. Supplementary Table S1, Supplementary Figure S1, Supplementary Figure S2, Supplementary Figure S3, Supplementary Table S2 [file 13148_2023_1544_MOESM1_ESM.docx]

**Supplementary Table S1.** Number of significant probes for one-sample *t*-tests for individual values against one pooled data for each site (820,677) and the Top 100 sites by different definitions.

|  | *P* < 0.05 (%) | BH (%) | *P* < 9.0E^-08^ (%) | Bonferroni (%) |
| --- | --- | --- | --- | --- |
| All sites (820,677) | | | | |
| β-value |  |  |  |  |
| A1 | 393,604 (48.0) | 312,240 (38.0) | 1,211 (0.1) | 917 (0.1) |
| A2 | 401,872 (49.0) | 316,877 (38.6) | 373 (0.05) | 277 (0.03) |
| B1 | 401,330 (48.9) | 323,818 (39.5) | 790 (0.1) | 605 (0.1) |
| B2 | 427,949 (52.1) | 357,836 (43.6) | 1,012 (0.1) | 786 (0.1) |
| M-value |  |  |  |  |
| A1 | 395,439 (48.2) | 313,896 (38.2) | 778 (0.1) | 569 (0.1) |
| A2 | 400,051 (48.7) | 315,968 (38.5) | 350 (0.04) | 260 (0.03) |
| B1 | 401,481 (48.9) | 325,089 (39.6) | 628 (0.1) | 471 (0.1) |
| B2 | 427,922 (52.1) | 358,410 (43.7) | 1208 (0.1) | 894 (0.1) |
| Top 100 sites | | | | |
| β-value |  |  |  |  |
| A1 | 44 | 36 | NA | 17 |
| A2 | 50 | 39 | NA | 17 |
| B1 | 43 | 41 | NA | 19 |
| B2 | 62 | 55 | NA | 24 |
| M-value |  |  |  |  |
| A1 | 44 | 37 | NA | 16 |
| A2 | 50 | 39 | NA | 18 |
| B1 | 43 | 40 | NA | 20 |
| B2 | 62 | 54 | NA | 24 |

BH: Benjamini-Hochberg, Bonferroni correction used *P* < 6.09253E-08 (0.05/820,677)

**Supplementary Figure S1.** Spectrophotometric UV spectra (A) UV spectra of each pooled sample. (B) UV spectra of pooled samples. Blank: measured using the TE buffer.

**Supplementary Figure S2.** The sample layout was balanced for the experimental group ID (upper) and age (lower).

**Supplementary Figure S3.** Example of singular value decomposition (SVD) before and after ComBAT processing to remove the confounding effects of non-biological unwanted variables.

**Supplementary Table S2.** Comparison of the predicted cellular heterogeneity.

|  | Individual |  | Pooled |  |
| --- | --- | --- | --- | --- |
|  | Group A | Group B | Group A | Group B |
| CD8+T | 0.10 ± 0.03 | 0.10 ± 0.03 | 0.13 ± 0.01 | 0.10 ± 0.02 |
| CD4+T | 0.15 ± 0.04 | 0.15 ± 0.05 | 0.17 ± 0.03 | 0.18 ± 0.01 |
| NK | 0.06 ± 0.02 | 0.05 ± 0.03 | 0.04 ± 0.02 | 0.05 ± 0.01 |
| B | 0.09 ± 0.02 | 0.10 ± 0.02 | 0.10 ± 0.00 | 0.10 ± 0.01 |
| Monocytes | 0.07 ± 0.02 | 0.06 ± 0.01 | 0.06 ± 0.01 | 0.06 ± 0.01 |
| Neutrophils | 0.52 ± 0.08 | 0.53 ± 0.10 | 0.48 ± 0.03 | 0.51 ± 0.03 |

Data are presented as mean ± standard deviation.
